# Lessons learnt from the rapid implementation of reusable personal protective equipment for COVID-19 in Malawi

**DOI:** 10.1136/bmjgh-2021-006498

**Published:** 2021-09-12

**Authors:** Fumbani Limani, David Garley, Derek Cocker, Priyanka Patel, Pratiksha Patel, Stephen Gordon, Mulinda Nyirenda, Servace Sakala, Luis A Gadama, Queen Dube, Feggie Bodole, Kwazizira Samson Mndolo, Kelvin Mponda, Bridget Freyne

**Affiliations:** 1 Medicine, Queen Elizabeth Central Hospital, Blantyre, Malawi; 2 Medicine, Malawi-Liverpool-Wellcome Trust Clinical Research Programme, Blantyre, Malawi; 3 Respiratory Group, Liverpool School of Tropical Medicine, Liverpool, UK; 4 Ministry of Health, Queen Elizabeth Central Hospital, Blantyre, Malawi; 5 Department of Internal Medicine, University of Malawi College of Medicine, Blantyre, Malawi; 6 Hospital Information, Queen Elizabeth Central Hospital, Blantyre, Malawi; 7 Department of Obstetrics and Gynaecology, University of Malawi College of Medicine, Blantyre, Malawi; 8 Paediatric Department, University of Malawi College of Medicine, Blantyre, Malawi; 9 Nursing, Queen Elizabeth Central Hospital, Blantyre, Malawi; 10 Clinical Infection, Microbiology and Immunology, University of Liverpool, Liverpool, UK; 11 Paediatrics, Malawi-Liverpool-Wellcome Trust Clinical Research Programme, Blantyre, Malawi

**Keywords:** COVID-19, public health, infections, diseases, disorders, injuries

## Abstract

The SARS-CoV-2 pandemic has challenged health systems and healthcare workers worldwide. Access to personal protective equipment (PPE) is essential to mitigate the risk of excess mortality in healthcare providers. In Malawi, the cost of PPE represents an additional drain on available resources. In the event of repeated waves of disease over several years, the development of sustainable systems of PPE is essential. We describe the development, early implementation and rapid scale up of a reusable gown service at a tertiary-level hospital in Blantyre, Malawi. Challenges included healthcare worker perceptions around the potential of reduced efficacy of cotton gowns, the need to plan for surge capacity and the need for ongoing training of laundry staff in safety and hygiene procedures. Benefits of the system included increased coverage, decreased cost and reduced waste disposal. The implementation of a reusable cotton gown service is feasible, acceptable and cost-effective in tertiary centres providing specialist COVID-19 care at the height of the pandemic. This innovation could be expanded beyond low-income settings.

**Summary box**

- Components of reusable personal protective equipment (PPE) are recommended by the WHO where disposable items are not available or sustainable.
- Programmes which implement reusable PPE need to be underpinned by reliable supply, ongoing monitoring and evaluation, and robust contingency planning.
- Given the prospect of prolonged circulation of SARS-CoV-2 in low and middle-income countries (LMICs), reusable PPE is a sustainable, environmentally friendly option which supports local business.
- Studies are required to empirically prove the safety of reusable PPE and these findings may be generalisable beyond LMIC contexts.

## Introduction

Pandemic preparedness stresses the need to stockpile essential equipment. In early 2020, worldwide stocks of personal protective equipment (PPE) became scarce and shortages were widespread.^1^ Despite collaboration between international agencies and local governments, individual PPE items doubled in price early in the pandemic.^2^ Ministries of Health in low and middle-income countries (LMICs) were unable to make the necessary purchases. Uncertainty about PPE contributed to industrial action by Malawian healthcare workers in April 2020.^3^ In response, the WHO released guidance on alternative forms of PPE where disposable alternatives were not available.^4^ In Malawi, disposable gowns were available for private purchase in April 2020 at a unit cost of US$10–US$15 which presented an unacceptable drain on resources.

The pandemic experience in Asia and Europe highlighted the risk of occupational exposure to SARS-CoV-2 for healthcare workers.^5^ The risk of occupational COVID-19 infection is related to the nature of the exposure (ie, the level of aerosol generated) and the adequacy of PPE.^6^ Reusable cotton gowns are not fluid repellent. Theoretically, they may not offer equivalent protection against prolonged droplet exposure when compared with fluid-repellent versions but the clinical relevance of this is unknown. Faced with the possibility of service disruption due to the cost and limited availability of gowns, the hospital administration at Queen Elizabeth Central Hospital (QECH) in Blantyre, Malawi opted for a reusable gown service on 8 April 2020. In anticipation of a surge in hospital admissions, the reusable PPE service was scaled up to 2700 gowns per week, across seven departments over 15 weeks. The aim of this paper is to share lessons, costs and tools associated with this rapid implementation.

## The context

Malawi is a low-income country in southern Africa with per capita health expenditure of US$19.^7^ Eighty per cent of the 19 million strong population earn subsistence income, making effective lockdown impossible.^8^ State healthcare is delivered by primary health centres supported by district and central hospitals. The QECH in Blantyre is the largest hospital in Malawi and the district hospital for Blantyre with a referral population of 5.5 million.^9^ The hospital has 1200 beds and up to 6000 admissions per month across 10 clinical departments. In the era of COVID-19, QECH administration worked with the Blantyre District Health Office to provide tertiary care to patients with suspected or confirmed COVID-19 in isolation wards on site. Since the first case of COVID-19 was reported in Malawi on 2 April 2020, there have been 142 confirmed COVID-19-positive patients admitted to QECH. The annual budget for QECH is 5.5 billion Malawi kwacha (£5.6 million) and the Central Medical Stores Trust (CMST) is the authorised supplier of medical goods. Prior to 2020, CMST supplied up to 48% of QECH needs with the shortfall supplemented through private purchase or donations (QECH budget 2020/2021).

In the context of a worldwide shortage of PPE, several interventions aimed at reducing consumption of PPE items at QECH were initiated, including (1) home-based testing of contacts by mobile teams; (2) postponement of non-urgent hospital services and (3) promotion of rational PPE use. Despite these actions, both the cost and availability of PPE items posed a threat to supply. Disposable gowns and suits were the most expensive and hardest to procure PPE items and the hospital opted for a reusable gown service. The desirable system features were: low cost, rapid initiation, scope to upscale and the potential for long-term integration into the existing hospital laundry system.

## Pre-implementation phase

### Development of a standard operating procedure

**Figure 1:**
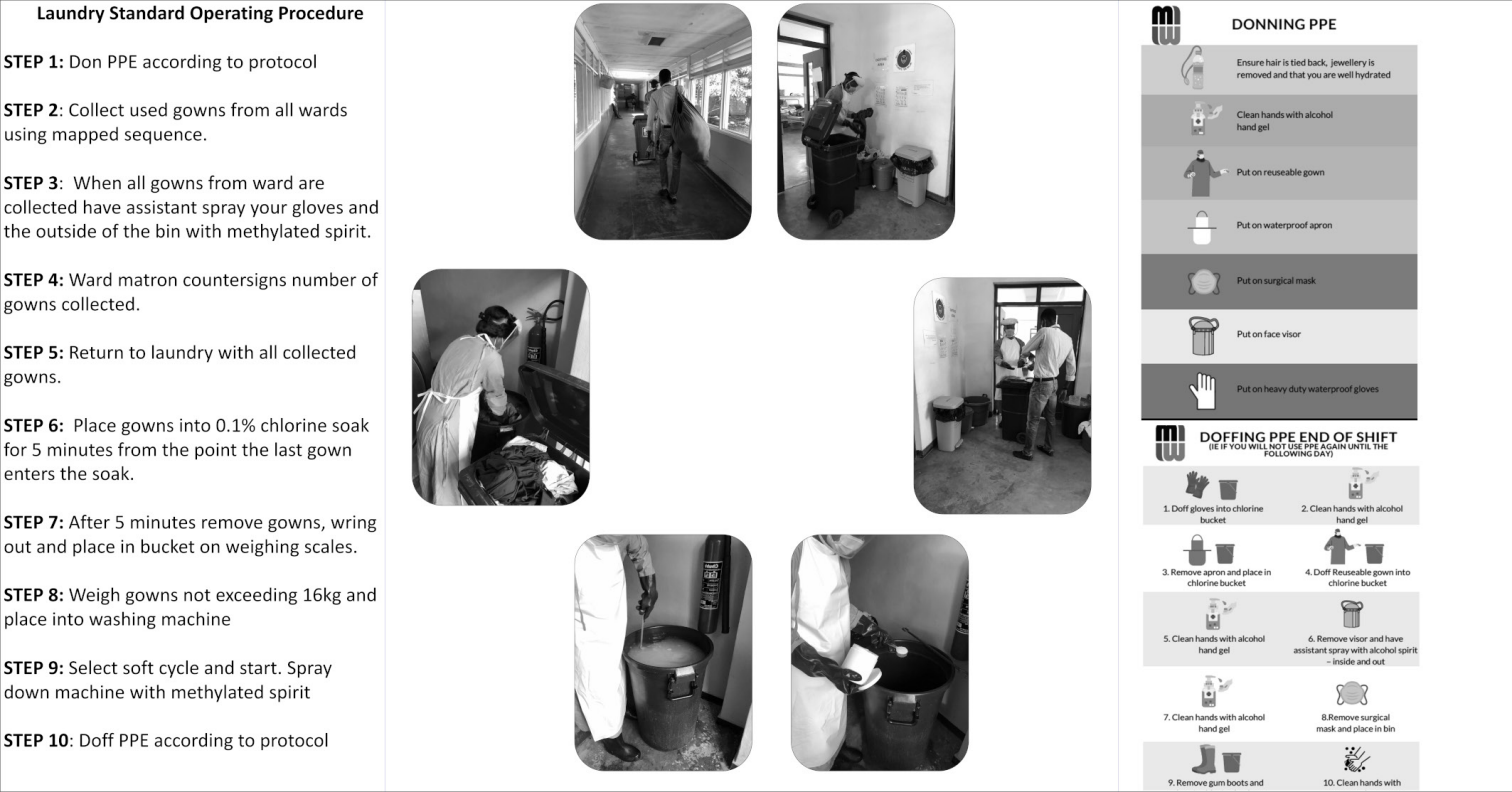
Overview of laundry SOP. Box 1: main steps in a full laundry cycle. Box 2: laundry team in QECH. Box 3: donning and doffing memory aids for laundry staff. All tools and materials provided in online supplemental material (images credited to Dr Fumbani Limani). PPE, personal protective equipment; QECH, Queen Elizabeth Central Hospital; SOP, standard operating procedure.

In the absence of specific guidance on sterilisation of reusable gowns, the WHO interim guidance on sterilisation of hospital linen in the era of COVID-19 was used.^4^ This document emphasises that chlorine sterilisation is less effective in the presence of organic matter. Ideally, linen should be washed, then soaked in chlorine and washed again. This procedure is time-consuming and requires substantial water and electricity, the cost of which increased by 50% during the pandemic (QECH budget 2020/2021). Discussions were held with nursing managers, health and safety officers, and representatives from the Departments of Medicine and Paediatrics on how to amend the procedure to promote feasibility and sustainability without compromising staff safety. Given that gowns covered with a plastic apron were typically not heavily soiled following a single shift, a decision was made to start with a 30-minute chlorine soak carried out at the ward doffing station, followed by a hot wash (figure 1 shows images of the laundry team members who gave consent for their images to be used).

10.1136/bmjgh-2021-006498.supp1 Supplementary data

Gown sterilisation on the ward was affected by two issues. First, when gowns were doffed directly into a basin of 0.5% chlorine, laundry staff were unable to confirm the minimum recommended soak time of 30 min, particularly where large numbers of gowns meant that submersion was not possible. Second, prolonged soaking prior to collection resulted in damage to the gowns. Taking this into account, the standard operating procedure (SOP) was revised, and ward staff were retrained to doff into mobile bins, allowing the laundry staff to collect the gowns and do the chlorine soak at the laundry with a stronger 0.1% chlorine solution for 5 min.

### Workspace

The main QECH laundry was running at capacity leaving existing hospital machines available only out of hours. The Malawi-Liverpool Wellcome Trust Research Programme had a furloughed laundry and an industrial drier previously associated with a paediatric research ward. This space along with a neighbouring disused storage room was recommissioned for this project. As capacity grew, a utility room in the Department of Obstetrics was added. This provided for a contingency laundry space and the potential for two teams to work in parallel during surge periods.

### Machinery

The choice of laundry machine is influenced by cost, availability and projected peak usage.

Using the WHO interim guidance on the rational use of PPE and our local staff numbers, we projected the quantity of gowns which would be required in frontline areas at peak turnover.^4^ We included all clinical and domestic staff from the adult and paediatric emergency departments, the COVID-19 isolation wards, the intensive care unit, the labour ward and operating theatres which resulted in a peak daily estimate of 250 gowns.

Following sterilisation in chlorine, gowns weighed 900 g giving an estimated requirement of 225 kg of laundry per day. To manage this volume of work, a large industrial machine was ideal but not locally available. To wash 225 kg of laundry in a day, an average 16 kg machine would need to run 14 times. At 45 min per cycle with a 10-minute changeover, this results in 10.5 hours per day. It was therefore decided to purchase two machines to shorten the laundry shift and avoid system failure in the event of machine malfunction.

**Figure 2:**
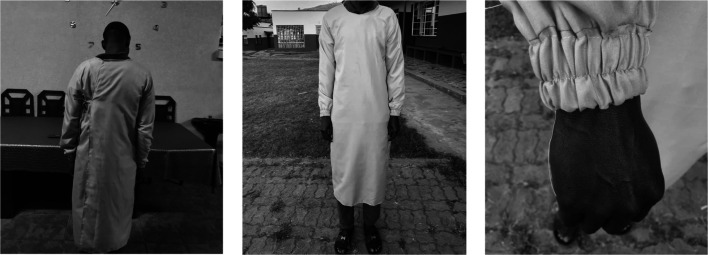
Details of the prototype reusable gowns (image credited to Dr David Garley).

Local tailors were approached to develop prototype gowns. The WHO guidance does not insist on fluid repellent material if a disposable plastic apron is used over the top of the gown. We chose close weaved polycotton as it is durable, low cost and widely available. Prototype gowns were presented to hospital managers and clinical staff for feedback. This exercise led to tightening the neck space, increasing the length and size, and loosening the elasticated wristbands (figure 2 shows a laundry team member who gave consent for their image to be used).

Six furloughed research fieldworkers were recruited to run the laundry. They worked alternate weeks in two teams. The initial project implementation was overseen by a team of six volunteers whose duties included maintaining the staff rota, linking the service with hospital departments, and coordinating PPE training sessions in all frontline areas to consolidate evidence-based use of PPE and allow for more accurate estimations of need prospectively. Laundry staff were trained on the SOP and the appropriate use of PPE through a combination of didactic and practical sessions over the course of 2 days.

## Implementation phase

The relatively slow progress of the COVID-19 pandemic in Malawi allowed a phased introduction of the laundry service over the course of 3 months (table 1). Early implementation was negatively affected by issues of acceptability and supply chain.

**Table 1:** Overview of gown usage weeks 1–11 of pandemic and % capacity used at each level of contingency

	M	T	W	T	F	S	S	Total	% L1	% L2	% L3
Week 1	5	12	5	15	23	2	10	72	6	3	2
Week 2	27	39	55	55	62	54	56	348	29	17	8.2
Week 3	56	82	67	66	75	61	44	451	38	21	11
Week 4	29	82	61	78	78	75	73	476	40	22	11
Week 5	79	90	105	133	97	69	58	631	53	30	15
Week 6	91	116	113	98	122	76	62	678	57	32	16
Week 7	107	102	119	143	161	132	140	904	76	43	22
Week 8	165	185	176	230	212	217	155	1340	113	64	32
Week 9	228	242	218	247	215	236	125	1511	127	72	36
Week 10	192	297	230	240	267	178	192	1596	134*	76	38
Week 11	223	347	291	359	274	243	219	1956	164*	93	47

L1=initial laundry capacity (170 gowns per day); L2=extended laundry hours (300 gowns per day) and L3=a second domestic washing machine (600 gowns per day).

*Instances where baseline capacity was exceeded.

### Acceptability

There were initial concerns from clinical staff about the relative safety of cotton gowns compared with disposable gowns or suits. This was particularly true in the emergency department, where the nature of the work made fluid repellent gowns preferable. Two strategies were employed to address these concerns. First, additional education sessions were arranged at a departmental level. During these sessions, it was acknowledged that reusable gowns were second line, and they were being deployed in the absence of a sustainable alternative. This validated staff concerns and provided a platform for further discussion. In addition, it was re-enforced that gowns were only one element of a system of PPE which needed to be kept intact in order to minimise infection. Thus, in the event of becoming soiled with bodily fluids, staff were advised to change the gowns and wash themselves. In addition, plastic aprons should be worn over the gown at all times to minimise this risk. Second, individual departmental heads were engaged by the hospital administration and the COVID-19 response team to address department-specific concerns.

### Gown supply and staff confidence

There were several instances of delayed access to gowns in clinical areas which was damaging to staff confidence in the service. To improve continuity of supply, a laminated poster with the phone and WhatsApp contact of the laundry coordinator was delivered to every ward and the matrons were given access to an emergency supply of gowns which could be deployed if necessary. These interventions were acceptable to clinical staff who called the laundry coordinator directly several times per week.

The importance of service continuity for staff confidence re-enforced the need for clear contingency planning around surge capacity and unexpected disruptions to the service. The baseline laundry capacity could process 170 gowns per day. Level 2 capacity involved extension of the hours of operation of the laundry with existing staff and had a capacity of 300 gowns per day (table 1, L2). Level 3 capacity involved the deployment of an additional domestic washing machine and had capacity for 600 gowns per day (table 1, L3). Table 1 illustrates how escalation to level 2 was required at week 8 following admission of the first positive case of COVID-19 to QECH, with full capacity in operation by week 11.

Additional contingency planning is outlined in table 2. The most common contingency plan deployed was the release of the buffer stock of gowns. Temporary use of the drier in the Department of Obstetrics and the need to line dry gowns and deploy buffer stock were also necessary during malfunction of the laundry tumble dryer.

**Table 2:** Contingency plans in place to support malfunction of any system elements

Event	Contingency
Washing machine malfunction	Procurement of 2nd and 3rd washing machine. Temporary out-of-hours usage of machine in the Department of Obstetrics agreed.
Drying machine malfunction	Drying lines identified with release of buffer gown stock. Temporary in-hours usage of the machine in the Department of Obstetrics agreed.
Reduced water supply	Washing machine manually loaded with bucket from alternative water source. Tank filling during hours of water availability.
Power cut	Use of generator. Drying via drying lines with release of buffer gown stock due to longer drying time.
Laundry staff worker contracts COVID-19	Team of three to self-isolate. Back-up furloughed staff completed laundry induction training in advance to allow immediate deployment. Back-up staffing by laundry supervisors as required.

## Monitoring and evaluation

To support safe and effective delivery of the service, two indicators were selected for prospective audit and feedback: the appropriate use of PPE by laundry staff and the adequacy of gown sterilisation. A cross-sectional audit of these indicators was done at week 6 of implementation, feedback was provided and re-audit occurred at week 8.

### Personal protective equipment

The correct use of PPE was essential for the safety of laundry staff. Observational audit (online supplemental appendix 2) was done to assess compliance with the SOP (online supplemental appendix 1). In addition, dedicated donning and doffing stations were established and equipped with memory aids (figure 1). Five episodes of donning PPE were observed over a 7-day period. The observed sequence of donning and doffing was checked against the sequence in the SOP including appropriate hand hygiene. For a single observation to be considered as correct, all steps needed to be complete. Initial fidelity with the SOP was low at 12.5% and was predominantly affected by inadequate hand hygiene. A refresher training session including a competency assessment was done and re-audit at week 8 showed improvement in fidelity to the SOP from 12.5% to 50% which resulted in the need for prospective review of procedures by supervisors. A third audit took place at 7 months and showed that PPE fidelity had increased to 60%.

### Sterilisation

The process for gown sterilisation is outlined in full in the laundry SOP (online supplemental appendix 1). In brief, a tablespoon of dry chlorine approximates the required weight to make 40 L of 0.1% chlorine. The chlorine is made up once per day. Batches of gowns are soaked for a minimum of 5 min to achieve sterilisation over the course of the day. Prolonged soaking can lead to damage. To audit the fidelity of this process, observations were done once for each team over a 7-day period. Data were collected on (1) the concentration of chlorine produced and (2) the duration of the soaks. All six staff members were assessed making chlorine and 100% of concentrations were within at least 0.01% of the correct minimum concentration. Four soaks were observed with 100% exceeding the minimum 5-minute duration and no soaks exceeded 7 min 40 s.

### Cost

Over the 8-week implementation phase, the local market cost for disposable gowns was £10.77 per unit. In contrast, the price of a reusable polycotton gown averaged £4.90 (£3.30–£6.60). With gown usage over those weeks at 4900 uses (from a total of 370 polycotton gowns in circulation), the total cost of disposable gowns would have been £52 773 compared with £1813 for provision of reusable gowns. Baseline infrastructural costs, staff wages, consumables and running costs are shown in online supplemental table 1 and detailed programme costings are available in online supplemental table 2. To estimate the crude cost–benefit of this service, we compared the costs of the reusable PPE system with the cost for disposable gowns at 2, 6 and 12 months (table 3). The unit price for disposable gowns has been adjusted down in line with local market values. The 2-month cost of the reusable service is relatively high as it includes all set-up costs, whereas subsequent evaluations include running cost only.

**Table 3:** Estimated savings provided by reusable gown service compared with disposable water repellent gowns

Month	Alternative PPE system costs	Cost of disposable gowns	Saving
2	24 997.75	52 773.00	27 775.25
6	35 177.75	158 319.00	123 141.25
12	50 447.75	316 638.00	266 190.25

The alternative system costs at month 2 include start-up costs while subsequent months are only running costs. The costs of gowns at 6 and 12 months have been adjusted for current market process.

PPE, personal protective equipment.

## Discussion

In terms of uptake, availability and cost-effectiveness, the system of reusable gowns was a success. Although there was a low incidence of occupational COVID-19 infection in QECH, we cannot draw conclusions on the relative safety of the reusable gown service. Overall, numbers of COVID-19-positive patients were low, the capacity for invasive aerosol-generating procedures is minimal and a limited supply of donated fluid repellent suits was deployed in the most high-risk areas when available. However, the overall effectiveness of a PPE system is a product of both PPE quality and coverage. The reusable PPE system provided full coverage of PPE to all frontline areas of the hospital which would not otherwise been possible, and which supported the uninterrupted provision of clinical services.

This implementation process was rapid and pragmatic with minimal capacity for evaluation which was limited to two process outcomes relevant to staff safety. Despite didactic and practical training sessions and the availability of memory aids and hand hygiene materials, adherence to PPE donning and doffing by laundry staff was very poor and only marginally improved by audit feedback. Interestingly, process outcomes related to procedural elements of the SOP were exceptionally well adhered to suggesting a significant behavioural element to poor adherence to PPE usage among staff. Given the implications for staff safety, regular audit of these activities including adaptations to improve adherence is essential to maintain a safe and reliable service.

Our experiences underline the importance of adequate and repeated stakeholder engagement to promote a system of alternative PPE use. Acceptability and uptake were improved through repeated education on the limitations of the system and transparency on the lack of a sustainable alternative. Departmental-level stakeholder engagement and open staff forums promoting debate on these issues improved uptake and acceptability and would be our key recommendation for sites hoping to implement a similar system.

Perhaps the most striking feature of the reusable gown service is the associated cost-savings for the hospital over a 12-month period even when adjusting for the stabilisation in unit cost of disposable gowns over time. The overall cost-saving, although substantial, is likely underestimated due to the lack of accurate hypothetical costs for procurement and distribution. Increased use of disposable PPE has resulted in increased medical waste around the world which has frequently overloaded municipalities’ capacity to safely dispose of it.^10^ The disposal of plastic waste is already a major environmental challenge in Malawi and the available facilities at QECH for incineration of biohazardous waste are limited, providing an additional advantage to this system in both cost and sustainability.

This implementation was designed to be as rapid as possible. It is now clear that the COVID-19 pandemic will present ongoing challenges to hospitals in LMICs for several years. Although not immediately possible in our setting, this service would have benefited from initial implementation within existing hospital sterilisation and laundry services to avoid the need for transition. In hospital settings where existing structures can be upgraded from the outset, this is to be encouraged.

## Conclusion

A system for the provision and sterilisation of reusable gowns is feasible and cost-effective across multiple settings in a busy tertiary level hospital in Malawi. Regular monitoring and evaluation are key to ensure staff safety through adherence to SOPs. Early stakeholder engagement which acknowledges (1) staff concerns on the use of second-line PPE and (2) the autonomy and context-specific concerns of individual hospital departments is important in promoting uptake and should accompany early implementation. Studies to prove the definitive safety of alternative PPE compared with first-line options are required before their use could be recommended worldwide. However, the benefits of this system in terms of both cost and environmental sustainability are transferable to high-income settings and could provide an excellent example of the successful diffusion of an LMIC innovation.

## Data Availability

All data relevant to this study are included in the article or iuploaded as supplementary information.
