# Supplementary material for: Lessons learnt from the rapid implementation of reusable personal protective equipment for COVID-19 in Malawi
Source: BMJ Glob Health. 2021 Sep 12;6(9):e006498. doi: 10.1136/bmjgh-2021-006498 (PMC8438572; doi:10.1136/bmjgh-2021-006498)
Supplement: Supplementary data [file bmjgh-2021-006498supp001.pdf]

LESSONS LEARNED FROM THE RAPID IMPLEMENTATION OF REUSABLE PERSONAL PROTECTIVE EQUIPMENT IN MALAWI DURING THE ERA OF COVID-19

Supplementary Materials

Supplementary Appendix 1: Standard Operating Procedure

**STANDARD OPERATING PROCEDURE**	**MLW.SOP.COVID.001**
**MLW Laundering of Reusable Gowns**	
**Effective date: 20th May 2020**	

	**Name**	**Signature**	**Date**
**Author**<br>**Job Title**	David Garley<br>Clinician	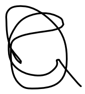	20/April/20
**Reviewer**<br>**Job Title**	Derek Cocker<br>Clinician	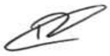	20/April/20
**Approver**<br>**Job Title**	Bridget Freyne<br>Clinician	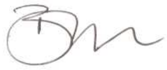	20/April/20

Review History

**Ver. No.**	**Reviewed by (Name)**	**Review date**	**Summary of changes**

BACKGROUND

COVID-19 is spread via respiratory droplet and fomite transmission. Personal protective equipment (PPE) is essential for protecting the work force from nosocomial infection, however there is a global shortage and adequate procurement of single-use PPE cannot be guaranteed. Transferring to reusable gowns will improve supply.

PURPOSE

This standard operating procedure (SOP) provides guidance to trained staff in the collection and safe transport of soiled PPE, its laundering, drying and redistribution.

RESPONSIBILITY

The facility supervisors are responsible for ensuring that staff are adequately trained in the practices and procedures outlined in this SOP and that these are observed. All staff members carrying out these procedures are responsible for understanding and carrying out the procedures exactly as described in this SOP.

PROCEDURE

Personnel requirements

- Six laundry staff
  - Four staff each day, seven days per week

Line management of laundry staff will be under the supervision of the MLW facilities department.

Materials needed

PPE – all laundry staff must wear the following PPE when collecting and laundering gowns

PPE

- Medical mask
- Face visor
- Gown
- Re-usable waterproof apron

- Long heavy duty re-usable waterproof gloves
- Gumboots

Laundry Service

- Wheelie bin
- Storage box with lid
- Metal trolley
- Clean laundry sack
- Weighing scales
- Bucket
- 70% ethanol
- Spray bottle
- 50L bin
- 0.1% chlorine
- PPE receipts
- Ledger
- Receipt box
- Pen
- Laundry powder
- Measuring scoop

Procedures

4.2 Collection of soiled PPE

1. Used gowns will be doffed into a 50L bin
2. Ensuring you are wearing appropriate PPE, draw the wheelie bin close to the 50L bin.
3. Lift gowns out of the bin and place carefully into the wheelie bin.
4. Repeat until all gowns are placed in the wheelie bin, counting as you go
5. Close the lid of the wheelie bin
6. Clean gloves and outside of wheelie bin with 70% ethanol
7. Have assistant fill in the PPE receipt with the number of gowns and have it counter signed by the nurse in charge
8. Repeat for all wards in the order 4a, screening tents, AETC, O&G, paeds A&E, paeds short stay, 3a, 3b, paediatric research ward
9. Transport the gowns to the laundry.
10. Transfer gowns into 50L bin containing 0.1% chlorine. The gowns must be fully submerged and soak for 5 minutes.
11. Record in ledger the number of gowns received from each ward, then store receipt in the box

4.3 Laundering gowns

1. Use the same PPE as for the collection of gowns
2. Put the bucket onto the scales and press ‘tare’ to ensure the display reads zero.
3. Ensuring gowns have soaked for a minimum of 5 minutes, carefully wring gowns out over the chlorine bucket and transfer to bucket, filling up to 8kgs, then tipping into laundry machine. Do this no more than twice so the maximum load of 16kgs is not exceeded. You may put less than 16kgs into the machine.

4. Load one scoop of laundry powder into the machine drawer, then close lid.
5. Select 'soft cycle', '40L' water, and press 'start'.
6. Doff PPE at the doffing station
7. Spray outside of machine and wheelie bin with 70% ethanol

4.4 Drying gowns

1. No PPE is required for this stage
2. Gather small armfuls of gowns from the laundry machine and carry to drier. Close door when complete, select cycle 17 and start machine.

4.5 Storing gowns

1. No PPE is required for this stage
2. Unload the gowns from the drier onto the metal trolley
3. Fold and store the gowns in the storage box or storage sacks

4.6 Redistributing gowns

1.Read from the ledger the number of gowns collected from each ward – this is the number that needs to be returned.

2.Count that number of gowns into the laundry sack and carry through to the ward.

3.Redistribute gowns to ward in the following order – paediatric research ward, 3a, 3b, paediatric short stay, O&G, AETC, screening tents, 4a.

4.7 Chlorine doffing bin

1.The chlorine in the laundry doffing bin must be made up each day to 0.1% concentration using the MLW chlorine preparation SOP.

4.8 Correct doffing of PPE and prevention of cross contamination

1. PPE must be doffed at the laundry doffing station. Additionally, the reusable heavy-duty gloves, aprons and gumboots should be soaked in the 0.05% chlorine rinse for 30 minutes, then rinsed in clean water and hung out to dry on the line. Visors should be sprayed with 70% ethanol.
2. Upmost care must be taken in maintaining a clean laundry, particularly due to its small space. Any cross contamination of clean gowns would have serious negative IPC outcomes.

HEALTH AND SAFETY

Ensure that Universal Precautions are used at all times

Risk assessment

**SOP for which the Risk Assessment is being done:**<br><br>Queen Elizabeth Hospital Laundering of Reusable Gowns

**Brief description of procedure:**<br><br>Collection of soiled gowns and their transport to the laundry. Laundering, drying and redistribution of gowns.	
**Cadre of staff carrying out the work:**<br><br>Laundry workers	
**Main hazards:**	**Precautions required:**
Risk of infection	Wear personal protective equipment at all times in line with MLW COVID-19 PPE policy.<br><br>Contamination incidents separately risk-assessed according to WHO criteria and in line with MLW COVID-19 PPE policy
Risk of contamination of laundry worker from soiled gowns during collection, transport and laundering.	Strict adherence to collection, transport and laundering instructions and immediate decontamination of spillages should they occur.
Risk of contamination of clean gowns leading to infection of healthcare workers	Strict adherence to laundry procedures with use of separate equipment for clean and soiled laundry.
**Special risks:**<br><br>-	**Precautions required:**<br><br>-
**Any spillage or other emergency procedures required?**	

Any and all spillage should be decontaminated with Virkon or 70% alcohol and removed with paper towel. Dispose of in designated biohazard waste bin.		
**Date for review of assessment:**		
**PI / Delegate:**	**Signed:**	**Date:**
**H&S Officer / Deputy:**	**Signed:**	**Date:**

ASSOCIATED DOCUMENTS

WHO COVID-19 PPE guidance

REFERENCES

MLW.SOP.HS.006. Chlorine preparation and use

ACKNOWLEDGEMENTS

Signing below indicates that I have read and understood this SOP, have been trained and will adhere to the processes contained in it.

Supplementary Appendix 2: Laundry Audit Plan and Results

Aim

To ensure the laundry service is introduced according to the standard operating procedure with all quality standards maintained, in particular those relating to staff safety and infection control.

	Objective	Target
1	To ensure correct PPE use by laundry staff	100%
2	To ensure correct minimum chlorine concentration	100%
3	To ensure gowns soaked for correct minimum amount of time	100%

Standards

MLW laundry standard operating procedure
MLW laundry PPE donning and doffing guide

Method

Objective 1:

Spot checks of laundry workers donning and doffing PPE to be carried out. Cross checking observed behaviour against a checklist of the correct sequence. Observations carried out once for each team during the week and once at the weekend.

Each laundry team is of three workers, one of whom don PPE for each collection. The one who dons PPE is rotated each time. The donning and doffing sequence will be recorded for up to four different collections. Doffing may be done differently depending on whether it is mid-shift and the PPE will be sprayed with ethanol and used again, or the last shift of the day, when the PPE is doffed into a chlorine soak. The two possible correct sequences are accounted for in the audit tool.

Objective 2:

In the SOP dry chlorine is measured out with a spoon and dissolved into 40L water to make 0.1% chlorine. As the chlorine is measured out it will be intercepted and weighed with an electronic balance. The weight should be correct for the volume of water used. Chlorine is made up once per day. Observations will be once for each team during the week and once at the weekend.

We will record the weight of chlorine measured out by each of the three team members – person A, B and C. Usually only one person weighs out the chlorine, so this is not the most natural observation, but will give us an idea of how accurate the entire team is at weighing out chlorine.

Objective 3:

Using 0.1% chlorine, gowns with potential COVID-19 contamination must be soaked for a minimum of five minutes to sterilise. If gowns are soaked for significantly longer then damage can be caused to the gowns. Gowns should start being removed from the soak from 5 minutes. The duration of soaks will be timed once for each staff team both during the week and at weekends.

While chlorine is made up once per day, there are multiple chlorine soaks throughout the day. Every soak in a day will be recorded.

Requirements:

1. 2 x observers
2. Audit tool
3. 1 x digital balance
4. 1 x stopwatch

Audit Tool

Checklist of objective observations to reduce inter-observer bias that cover the three objectives. Also include a white space area for general observations that do not directly relate to an objective but are still relevant.

Data

Audit observations were carried out over two days during the week and two days at the weekend.

	Objective	Target	Result
1	To ensure correct PPE use by laundry staff	100%	12.5%
2	To ensure correct minimum chlorine concentration	100%	100%
3	To ensure gowns soaked for the correct minimum amount of time	100%	100%

Analysis and conclusion

1) Nine donning procedures were observed, 2 (22%) completed all steps correctly. Of the remaining seven none used appropriate hand hygiene but three otherwise followed the correct steps. Four neither cleaned their hands first nor followed the correct steps.

2) Eight doffing procedures were observed. One (12.5%) was carried out with all steps in the correct order. Of the seven, four failed by not completing hand washing steps and the remaining two neither cleaned their hands nor followed the correct steps.

3) All six staff were audited on the chlorine concentration they made. In 100% cases the concentration was within 0.01% of the correct concentration

4) Eight chlorine soaks were observed and 100% were over 5 minutes. Some soaks went on as long as 26 minutes.

In both donning and doffing the commonest problem is not cleaning hands, either with alcogel before and during, or with soap and water at the end.

The chlorine solution is consistently made to the correct concentration

Soaks are carried out for the minimum time but should be stopped by transferring gowns to a water rinse to prevent further damage to gowns.

Intervention planning

1) Donning and doffing refresher course
2) Introduction of a separate water rinse once 5-minute chlorine soak is complete

Re-audit

	Objective	Target	1st Audit	2nd Audit
1	To ensure correct PPE use by laundry staff	100%	12.5%	50%
2	To ensure correct minimum chlorine concentration	100%	100%	100%
3	To ensure gowns soaked for the correct minimum amount of time	100%	100%	100%

1) Five doffings were observed, 4 (80%) carried out all steps correctly. One did not wash hands prior to starting donning.

2) Four doffings were observed, 2 (50%) carried out all steps correctly. One did not spray their gloves before removal, and one did not clean hands their hands with soap and water at the end

3) All six staff were assessed making chlorine and 100% concentrations were within at least 0.01% of the correct minimum concentration

4) Four soaks were observed with 100% exceeding minimum 5-minute duration. No soaks exceeded 7m40s.

Third Audit

While the results of the second audit showed improvement in PPE use, the results were still below the standard of 100%. Objective 1 and 3 were re-audited.

The reaudit results are as follows:

	Objective	Target	1st Audit	2nd Audit	3rd Audit
1	To ensure correct PPE use by laundry staff	100%	12.5%	50%	60%
2	To ensure correct minimum chlorine concentration	100%	100%	100%	N/A
3	To ensure gowns soaked for the correct minimum amount of time	100%	100%	100%	100%

1) Five donning procedures were observed, three of which were carried out entirely correctly. One donning had 4 out of 6 steps correct, and one had 5 out of 6 steps correct.

2) Five doffing procedures were observed, all of which were carried out with all 12 steps correct.

3) Five soaks were observed, all of which were over the minimum time.

	Target	1st Audit	2nd Audit	3rd Audit
Donning	100%	22%	80%	60%
Doffing	100%	12.5%	50%	100%

The results of the first objective have been broken down into donning and doffing.

Supplementary Material 3: Laundry Audit tool

Date:
David Garley/Fumbani Limani

Objective 1

Donning								
Correct Order	Person A	Person B	Person A	Person B	Person A	Person B	Person A	Person B
Clean hands								
Gown								
Apron								
Mask								
Visor								
Gloves								
Number correct								

Doffing								
Correct Order	Person A	Person B	Person A	Person B	Person A	Person B	Person A	Person B
Mid/end doff								
Spray gloves								
Remove gloves								
Clean hands								

Spray/soak apron								
Doff gown								
Clean hands								
Remove and spray visor								
Clean hands								
Remove mask								
Doff gumboots and spray/soak								
Wash hands soap/water								
Number correct								

Objective 2

Staff member	Weight of chlorine	Volume of water	Conc of chlorine
Person A			
Person B			
Person C			

Objective 3

Load Number	Time	Load Number	Time

Additional Comments

**Start-up costs**				
**Category**	**Item**	**Quantity**	**Unit Cost (£)**	**Price (£)**
Machines and Maintenance	16kg washing machine	2	480	960
	Industrial washing machine	1	15,622	15,622
	Initial machine servicing	1	100	100
	**Sub Total**			**16,682**
General Equipment	160L wheelie bins	2	480	960
	Wall mounted gel dispenser	1	50	50
	Plastic storage box	1	25	25
	50L bin	2	100	200
	20L bin	1	5	5
	Tailored laundry sacks	3	6.25	18.75
	Plastic scoops	1	5	5
	Stirring rod	1	0.5	0.5
	Mop	2	3	6
	Broom	1	2.5	2.5
	Spray bottles	2	1.50	3
	**Sub Total**			**1,275.75**
Reusable PPE	Overalls	6	13	78
	Heavy duty gloves	5	1.8	9
	Heavy duty aprons	4	3	12
	Gumboots med/large	5	6	30
	Gumboots small	1	8	8
	Gowns	370	4.9	1,813
	**Sub Total**			**1,950**
**Start-up Total**				**19,907.75**
**Monthly running costs (total 6 months)**				
Sterilisation	Washing powder	6kg	6	36
	Chlorine	8kg	29	174

	Ethanol	6L	4	24
PPE & hand hygiene	Alcohol Hand Gel	1.5L	30	180
	Hand soap	100ml	1	6
	Non-sterile gloves	150	10	60
	Medical masks	75	40	240
Maintenance	Machine maintenance	1	100	600
	General maintenance	1	35	210
	Gown repair	50	245	1,470
**Sub total**				**3,000**
**Staff costs (total 6 months)**				
Wages (6 staff)		1 month	1830	10,980
Transport allowance (6 staff)		1 month	215	1,290
**Sub Total**				**12,270**
**Total**				**15,270**

Suuplementary Table 1: Itemised start-up and running costs for laundry service for 6 months.
